# Protein analysis by time-resolved measurements with an electro-switchable DNA chip

**DOI:** 10.1038/ncomms3099

**Published:** 2013-07-10

**Authors:** Andreas Langer, Paul A. Hampel, Wolfgang Kaiser, Jelena Knezevic, Thomas Welte, Valentina Villa, Makiko Maruyama, Matej Svejda, Simone Jähner, Frank Fischer, Ralf Strasser, Ulrich Rant

**Affiliations:** 1 Chemistry Department and Walter Schottky Institute, Technische Universität München, Lichtenbergstrasse 4, 85748 Garching, Germany

## Abstract

Measurements in stationary or mobile phases are fundamental principles in protein analysis. Although the immobilization of molecules on solid supports allows for the parallel analysis of interactions, properties like size or shape are usually inferred from the molecular mobility under the influence of external forces. However, as these principles are mutually exclusive, a comprehensive characterization of proteins usually involves a multi-step workflow. Here we show how these measurement modalities can be reconciled by tethering proteins to a surface via dynamically actuated nanolevers. Short DNA strands, which are switched by alternating electric fields, are employed as capture probes to bind target proteins. By swaying the proteins over nanometre amplitudes and comparing their motional dynamics to a theoretical model, the protein diameter can be quantified with Angström accuracy. Alterations in the tertiary protein structure (folding) and conformational changes are readily detected, and even post-translational modifications are revealed by time-resolved molecular dynamics measurements.

## Abstract (web summary)

The comprehensive bioanalysis of proteins usually requires multi-step surface and mobile phase measurements. Here, the authors use chips functionalized with dynamically actuated nanolevers—DNA strands that can be switched in an electric field—to obtain motional dynamic measurements of proteins on a chip.

## Graphical abstract

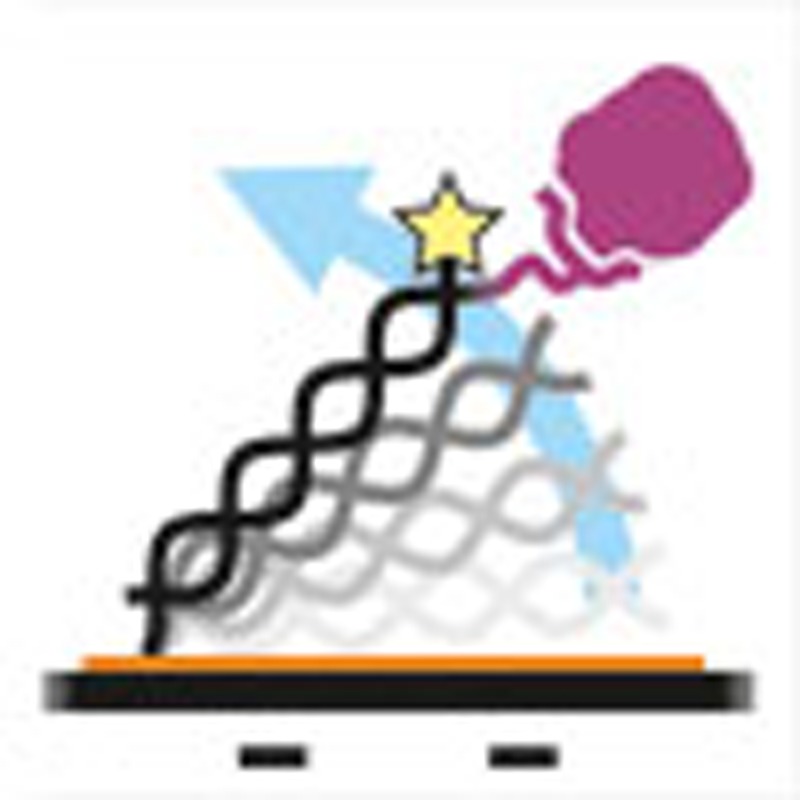

Powerful methods for the analysis of proteins and their interactions with small molecules, nucleic acids or other proteins are a cornerstone in the development of new drugs and next-generation disease diagnostics^1^. Commonly, molecular interactions are analysed with surface biosensors, which measure the association/dissociation of solute target molecules to/from surface-immobilized capture molecules. State-of-the-art platform technologies like surface plasmon resonance systems^2^,^3^, quartz crystal microbalances^4^, surface acoustic wave sensors^5^ or biolayer interferometry^6^ provide kinetic rate constants (*k*_on_, *k*_off_) and dissociation constants (*K*_D_). Biosensors with nanoscale features often outperform their macroscopic counterparts with respect to improved detection limits and minimal sample consumption^7^,^8^,^9^. Yet, these methods do not yield reliable information about protein size and shape (folding state), or molecular aggregation. In particular, chemically subtle changes, such as post-translational modifications, remain undetected. As the biological functionality of a protein inevitably depends on its structure, it is essential to determine these critical parameters in complementary assays, but gel electrophoresis, dynamic light scattering (DLS), pulsed NMR, circular dichroism (CD) or liquid chromatography, still require large amounts of sample and often cannot be performed under native conditions. Thus, when screening for biological drug candidates (therapeutic proteins)^1^,^10^, it remains a challenge to integrate a combination of methods in a high-throughput workflow that generates the required information content.

A promising route toward this goal is to employ stimuli-responsive molecular layers^11^,^12^, which provide added functionality compared with the passive surface coatings used in conventional biosensors. Following the discovery of the electrically induced conformation switching of DNA oligonucleotides^13^ on metal surfaces and ensuing fundamental investigations^14^,^15^,^16^,^17^, electrically switchable biosurfaces have been used successfully for the detection of DNA and protein targets with high sensitivity^18^,^19^,^20^,^21^. Frequency-resolved experiments have highlighted the potential to extract information from the induced molecular motion^14^,^22^, but because of the lack of time-resolved (TR) data and theoretical models, this measurement modality has not proven itself in an application-oriented setting so far.

Here we investigate the molecular dynamics of proteins, which are set in motion by electrically actuated DNA levers. We introduce a TR measurement technique and a theoretical model, which make it possible to detect changes in the protein structure and to quantitatively determine a protein’s diameter. Simultaneously, kinetic rate constants and dissociation constants are obtained. The method does not require labelling of analyte molecules, uses a parallel microelectrode format for multiplexed assays and microfluidics for low sample consumption.

## Results

### TR DNA switching and protein detection

**Figure 1:**
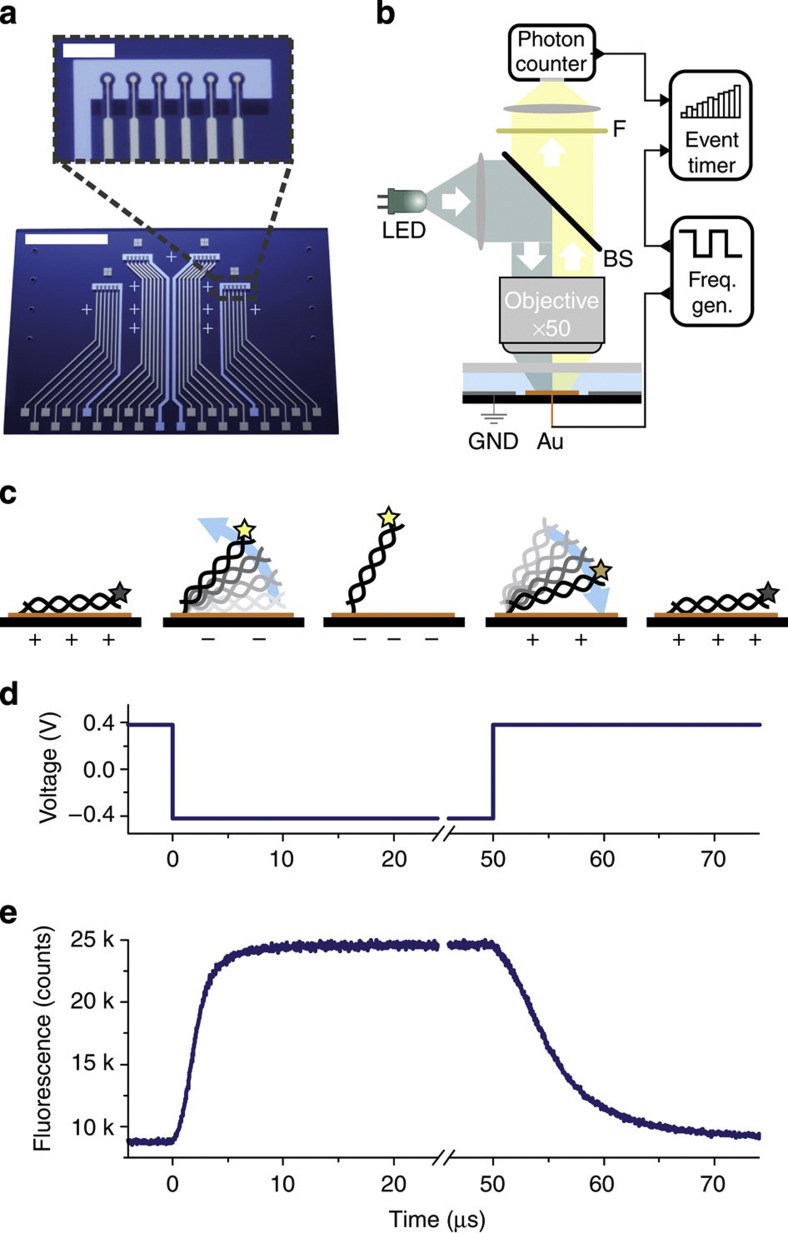
Time-resolved DNA switching measurement. (**a**) The biochip features 24 circular Au microelectrodes (∅=120 μm), which are individually addressable with different receptors for target molecules via DNA-directed assembly^44^. Six microelectrodes are arranged together with one Pt counter electrode in four flow channels (not shown), which are connected via eight holes in the glass substrate. White scale bars, 10 mm and 1 mm (inset). (**b**) An epifluorescence setup is used for the optical detection of DNA orientation in real time. A frequency generator supplies alternating voltages to the electrodes. This actuates the DNA levers and simultaneously triggers an event timer that records single photons emitted by the fluorescently labelled DNA. (**c**) Schematic of the electrically induced DNA switching. The fluorophore emission (depicted as a star) is quenched in close proximity to the surface. (**d**) Square wave voltage, which is applied to the Au electrodes, versus Pt (10 kHz) and (**e**) time-resolved fluorescence response of the 48 bp Cy3-labelled DNA layer.

Double-stranded DNA oligonucleotides (48 bp) are end-tethered via thiol chemistry to gold microelectrodes, which are arranged in sextuplets within four individually addressable flow channels (*V*=1 μl) on a glass substrate (Fig. 1a). An epifluorescence setup (Fig. 1b) is used to measure the orientation of the DNA levers with respect to the surface. The fluorescence from Cy3 dyes attached to the DNAs’ distal ends is gradually quenched when the DNA levers tilt toward the gold surface, due to a non-radiative energy transfer by near-field interactions with the metal^23^. This allows gauging the distance of the DNAs’ upper ends to the surface and monitoring changes in the DNA orientation in real time.

Alternating potentials that are applied to the electrodes repel (or attract) the negatively charged DNA and hence switch the lever orientation between a standing and a lying state (Fig. 1c and Supplementary Fig. S1). Generally, special care must be taken to prepare low-density DNA layers so that the molecules are not hampered by steric interactions^14^; here, this is accomplished by adjusting the DNA density through electrical desorption (see Methods). Employing time-correlated single photon counting, we are able to resolve the upward and downward motion of the DNA levers with 32 ns precision. The delay time of the arrival of a photon at the detector is recorded with respect to the edge of the applied square-wave voltage pulse by an event timer. A fluorescence histogram of satisfactory signal-to-noise ratio is acquired within seconds when driving the ~10^6^ DNA levers, which are present on one electrode at 10 kHz (Fig. 1d).

**Figure 2:**
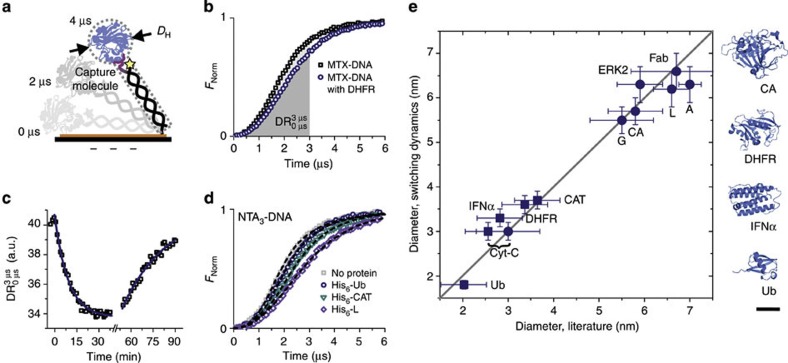
Protein detection and size analysis. (**a**) Schematic of a DNA lever with a protein (effective protein diameter *D*_H_) bound via a capture molecule (purple), which is covalently attached to one single strand. Dashed lines indicate the ‘Lollipop’ silhouette used for the calculation. (**b**,**c**) Binding of the enzyme DHFR (21 kDa) to the anticancer drug MTX, which is attached to the DNA’s top end. (**b**) Fluorescence response during upward switching for MTX-modified DNA without and with bound DHFR. Areas under the curves denote the DR between 0 and 3 μs, which is a parameter gauging the swiftness of the motion. (**c**) Real-time binding and dissociation of 10 nM DHFR to MTX-modified DNA levers. The solid lines are single exponential fits to the data. Kinetic rate constants of *k*_on_=(1.25±0.09) · 10^5^ M^−1^ s^−1^ and *k*_off_=(5.7±1.0) · 10^−4^ s^−1^ are obtained, yielding a dissociation constant of *K*_D_=(4.6±0.8) nM. (**d**) Upward switching fluorescence response for bare DNA and three DNA–protein complexes. The His_6_-tagged proteins ubiquitin (Ub, 8.5 kDa), chloramphenicol acetyl transferase (CAT, 29 kDa) and protein L (43 kDa) were captured via NTA_3_-tagged DNA. Dashed lines are calculated with the theoretical Lollipop model. (**e**) Comparison of protein diameters analysed from switching dynamics experiments using the Lollipop model with DLS (circles) or literature values from X-ray diffraction structure data (squares). Cytochrome-C (Cyt-C, 12 kDa), interferon-α (IFNα, 18 kDa), carbonic anhydrase (CA, 29 kDa), IgG-binding recombinant proteins A (42 kDa) and G (26 kDa), protein kinase ERK2 (ref. 45) (42 kDa), Fab fragment (50 kDa). Representative structures of four proteins with *D*_H_≈2–6 nm are drawn to scale on the right. Black scale bar, 2 nm.

By attaching designated capture molecules to the DNAs’ top ends, the levers can specifically bind target proteins from solution (Fig. 2a). As a consequence of the protein binding, the DNA lever motion slows down. This is shown at the example of the small enzyme dihydrofolate reductase (DHFR, 21 kDa) binding to DNA levers, which have been modified with methotrexate (MTX), an anticancer drug that inhibits DHFR (Fig. 2b). In fact, binding kinetics can be followed in real time, and the association and dissociation rates *k*_on_ and *k*_off_, and the dissociation constant *K*_D_ can be determined as shown in Fig. 2c and in more detail elsewhere^24^. For optimal signal-to-noise ratios at high sampling rates, it is practical to define a ‘dynamic response’ (DR) parameter, which equates to the area under the fluorescence curve when switching upward (Figs 2b and 4b), or the area above the curve when switching downward, respectively (Fig. 4d), *cf.* equation (1). With this definition, DR corresponds to the change in height (in fluorescence units) that is covered by the top end of the DNA lever within a given time period. High DR values indicate fast switching. We also note that not unexpectedly, the switching dynamics depend on the DNA lever length. The DR decreases with increasing DNA length (that is, a higher rotational friction coefficient of the DNA strand), as will be reported for oligos up to 72 bp in more detail elsewhere.

A particular advantage of this method is that the signal is not sensitive to changes in the absolute fluorescence intensity emitted by the switching DNA layer or to background fluorescence. Autofluorescence, which may arise from a highly concentrated protein solution above the sensor, for instance, may easily be discriminated (and subtracted) from the genuine signal, because contrary to the DNA switching it is not modulated synchronously with the applied ac potential. Even the degradation of fluorophores (for example, by photobleaching) does not compromise the validity of the dynamics measurement, as the switching behaviour of the remaining fluorescent DNA strands remains unaltered. In the case of significant signal loss, however, the acquisition time (number of integrated switching cycles) needs to be increased to maintain a satisfying signal-to-noise ratio.

### Determination of the hydrodynamic protein diameter

In experiments with differently sized proteins we found that the switching dynamics of DNA–protein complexes gradually slow down with increasing protein size (Fig. 2d). Hence, we surmised that by calculating the DNA–protein motion, it should be possible to infer the hydrodynamic diameter of the bound protein.

To this end we developed an analytical model, which treats the protein as a (potentially charged) sphere on top of a charged DNA rod (Fig. 2a), resembling the shape of a lollipop. Depending on the surface potential ϕ of the microelectrode, each DNA orientation features a distinct free energy *G*(*α*), which only depends on the out-of-plane angle *α*: *G*(*α*)=*U*(*α*)–*TS*(*α*). The first term describes the electrostatic energy of a charged DNA rod and a (charged) protein within a screened, exponentially decaying electric surface field, whereas the second term derives from the entropy of a rod, which is capable of rotating around a pivot point at one end. Detailed expressions are given in equations 2 and 3 in the Methods section. In equilibrium, the ensemble of DNA molecules adopts a Boltzmann probability distribution of angular orientations: *p*(*α*)~exp{–*G*(*α*)/k_B_*T*}. The temporal evolution of *p*(*α*,*t*) in a time-variable surface potential can be calculated from the Fokker-Planck drift-diffusion equation (equation 4), which contains the rotational diffusion coefficient *D*_r_^comp^ of the DNA–protein complex as the essential free parameter that needs to be determined. This is done by transforming the *p*(*α*,*t*) distributions to measurable fluorescence intensities with the known distance dependence of the fluorescence quantum yield^23^ (equation 5) and fitting the calculations to the experimental data. By comparison with the rotational diffusion coefficient of bare DNA (*D*_r_^DNA^), the contribution of the protein to the total rotational friction is obtained. Using a modified Stokes–Einstein equation (equation 6) yields the effective protein diameter *D*_H_, which corresponds to the diameter of a sphere with the same hydrodynamic friction as the investigated protein.

Analysis of experimental TR switching curves with the ‘Lollipop’ model enables the determination of absolute values of a protein’s effective hydrodynamic diameter. It should be emphasized that the motion of the DNA–protein complex through the aqueous solution is not a resonant oscillation, but that the movement is strongly damped. Therefore, hydrodynamic friction but not molecular mass limits the switching and the dynamics are governed by the protein size and shape. The calculated fluorescence curves agree very well with the experimental results (Fig. 2d and Supplementary Fig. S2). Eleven different proteins with diameters ranging from 2 to 7 nm (molecular weights of ~8–50 kDa) were analysed and compared with complementary DLS measurements and literature X-ray diffraction data (Fig. 2e). The evaluated *D*_H_ diameters are in very good agreement with data from complementary methods and, on average, deviate by less than 0.3 nm from the calibration line.

### Mixture and conformation analysis

**Figure 3:**
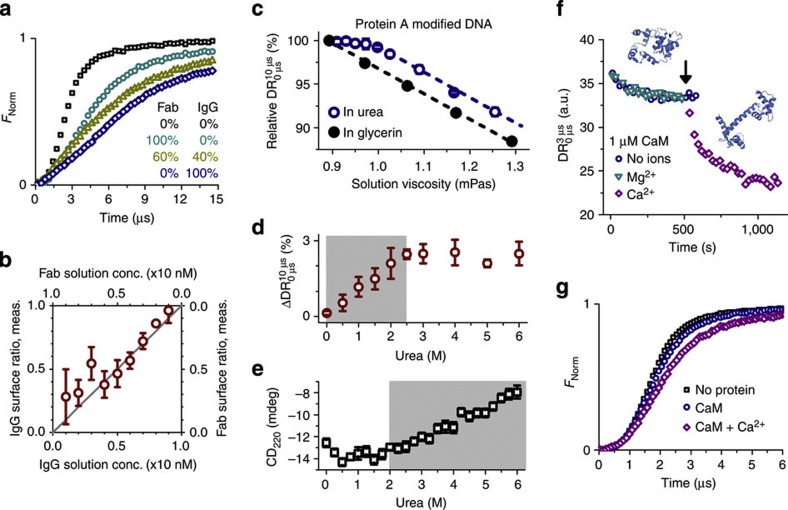
Mixture and conformation analyses. (**a**,**b**) Mixture analysis of antibody solutions. (**a**) Time-resolved upward motions of biotinylated DNA levers before and after binding antibiotin full IgGs and IgG fragments (Fab) from solutions containing different mixing ratios. (**b**) Comparison of measured and preadjusted mixing ratios. Data were analysed from DR values evaluated between 3 and 9 μs. (**c**–**e**) Unfolding of protein A. (**c**) Relative DR values for protein A-modified DNA levers in urea (which acts as a denaturation agent) and glycerin (as a negative control) as a function of solution viscosity. Values are normalized by the DR in urea- and glycerin-free buffer. Lines are parallel linear fits. All errors for glycerin are smaller than the symbols. (**d**) Differential analysis of protein A-modified samples reveals the disintegration of tertiary protein structure (shaded region). (**e**) Circular dichroism measurements at 220 nm indicate unfolding of secondary structures of protein A at urea concentrations above 2 M. (**f**,**g**) Conformation analysis of CaM. (**f**) 1 μM His_6_-tagged CaM is bound to NTA_3_-modified DNA. Ion-free CaM (blue circles) and CaM mixed with 100 μM MgCl_2_ (cyan triangles) show identical binding curves, whereas the addition of 100 μM CaCl_2_ (black arrow, purple diamonds) causes an additional decrease in DR. (**g**) Comparison of the fluorescence response during the upward switching cycle for saturated layers of Ca^2+^-free CaM and CaM with bound Ca^2+^. All error bars represent s.d. derived from measurements on three electrodes.

Given that proteins of diverse size can be clearly discriminated according to their switching dynamics, we went on to address the question whether the simultaneous occurrence of different fragmentation (or aggregation) states of a protein can be quantified with a surface biosensor. As an exemplary use case in an antibody engineering setting, whole antibiotin IgGs (150 kDa) were mixed with IgG fragments (Fab, 50 kDa) at different ratios totalling at a concentration of 10 nM (>>K_D_). After incubating biotinylated DNA levers with these mixtures, the switching dynamics were analysed (Fig. 3a). The DR was found to depend linearly on the [IgG]:[Fab] mixing ratio. Hence, by linear interpolation of the measured DR values between the 
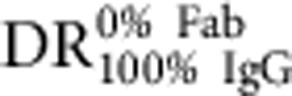
 and 
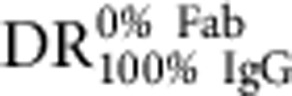
 values, the [IgG]:[Fab] ratios could be determined on the surface (Fig. 3b). The relative abundance of the two IgG species in solution could be quantitated from the surface measurement with an error of 10%.

The switching dynamics measurement also directly reveals conformational changes in the protein structure. To investigate unfolding processes, protein A (*S. aureus*, 42 kDa), a cell wall protein that enables bacteria to evade an immune response by binding the Fc region of IgG antibodies, was covalently coupled to DNA and the protein activity was verified (Supplementary Fig. S3). Urea was used as a chaotropic agent and glycerin as a negative control. As expected, the DR decreases with increasing solution viscosity, because hydrodynamic friction increases. For unmodified DNA, the viscosity dependence is linear and exactly the same for glycerin and urea solutions, confirming that this is indeed a pure viscosity effect (Supplementary Fig. S4). Protein A-modified DNA, by contrast, exhibits a non-linear behaviour only when exposed to the denaturation agent urea (Fig. 3c).

A transition regime in the urea concentration range from 0 to 2.5 M becomes clearly apparent (Fig. 3d) after correcting the viscosity effect by subtracting the linear response of the negative glycerin control. We attribute this transition to the unfolding of the protein’s tertiary structure. As the protein conformation transforms from a native globular structure to a denatured polypeptide chain, its hydrodynamic friction decreases and consequently the viscosity-corrected motion of the DNA–protein complex accelerates. It is interesting to compare this result with circular dichroism (CD) experiments, which indicate denaturation at higher urea concentrations, that is, >2 M (Fig. 3e and Supplementary Fig. S5). This is because of the fact that CD is mainly sensitive to the decay of secondary structures, which are preserved even when the tertiary structure has already disintegrated and the protein exists in an intermediate ‘molten globule’ state^25^. Similar results were reported in protein unfolding studies with electrospray ionization mass spectrometry^26^. Complementary to these techniques, the switching method is well suited for detecting minor deviations in the tertiary protein structure, which is particularly important as changes in the tertiary structure generally entail changes in—or the loss of—protein activity.

Conformational changes in the protein tertiary structure may also originate from the binding of small ligand molecules or ions. A well-studied example is calcium-modulated protein (calmodulin, CaM), a calcium-binding messenger protein in eukaryotic cells. Upon calcium binding (*K*_D_=1–10 μM^27^,^28^,^29^) (refs 27,28,28), CaM changes its structure from a globular to a dumb-bell-shaped form by rearranging hydrophobic methyl groups from methionine residues (*cf.* pdb entries 1QX5 and 1CLL)^30^,^31^. We were able to detect this conformational change with the switching dynamics measurement. Figure 3f shows the binding of 1 μM His_6_-tagged CaM to a layer of NTA_3_-modified DNA. Identical binding curves are obtained in the absence as well as in the presence of Mg^2+^ ions, which should not bind to CaM^27^,^28^,^29^ and are used here as a negative control for ion specificity. When flowing a 100 μM CaCl_2_ solution across the surface, the DR decreases as Ca^2+^ ions are incorporated into CaM. As the dumb-bell-shaped Ca^2+^-bound form of CaM effectuates a higher hydrodynamic friction than the Ca^2+^-free apoconformation^32^, the switching motion slows considerably (Fig. 3g and Supplementary Fig. S6).

### Detection of post-translational modifications

To assess the sensitivity of the switching dynamics measurement with regard to the detection of subtle chemical changes in a protein, we investigated two post-translational modifications: the glycosylation of the β-subunit of human chorionic gonadotropin (hCGβ), a hormone produced during pregnancy, which is also associated with some forms of tumours, and the phosphorylation of the extracellular signal-regulated protein kinase ERK2 (MAPK1), which is—among many biochemical processes—for instance, involved in transcriptional regulation.

**Figure 4:**
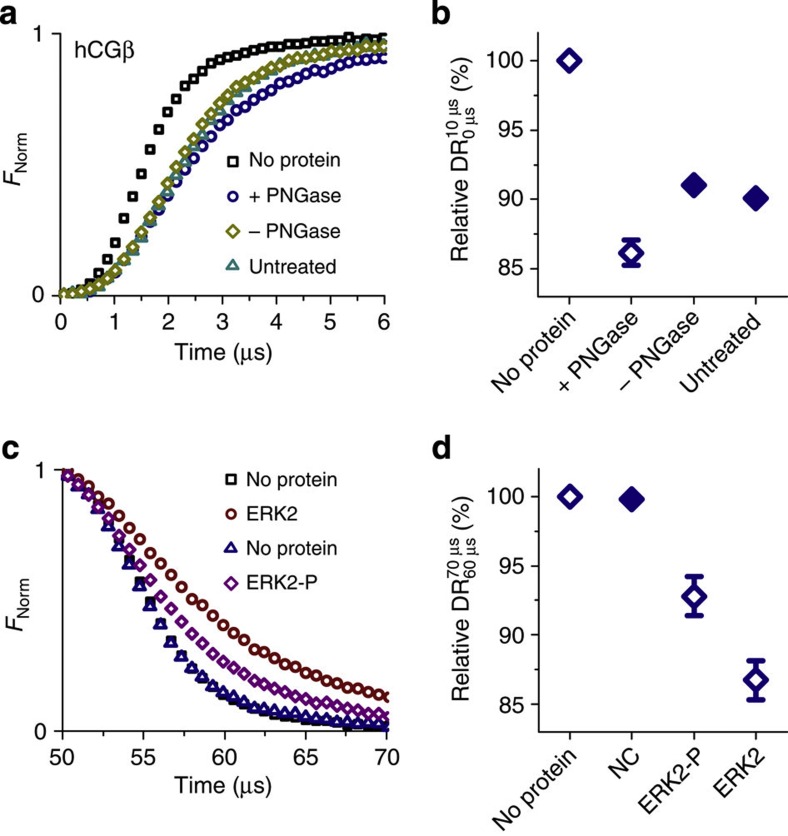
Post-translational modifications. (**a**,**b**) Detection of the deglycosylated state of the protein hCGβ. (**a**) Upward switching fluorescence response for conjugates of DNA with deglycosylated (+PNGase) and glycosylated forms (−PNGase, untreated) of hCGβ. (**b**) Average upward DR values for the conjugates (*n*=3). (**c**,**d**) Detection of the phosphorylation state of the protein kinase ERK2. (**c**) Downward switching fluorescence response of NTA_3_-tagged DNA levers before and after binding His_6_-tagged ERK2 and phosphorylated ERK2-P. (**d**) Average downward DR values of unmodified (negative control, NC) DNA levers and NTA_3_-tagged DNA levers after exposure to ERK2 and ERK2-P, respectively (*n*=6). Values are normalized by the DR of bare DNA levers.

The hCGβ was covalently conjugated to DNA in its native glycosylated and its non-glycosylated state; the latter was prepared by cleaving glycans from native hCGβ with the glycosidase peptide-*N*-glycosidase (PNGase). The different glycosylation states can clearly be discriminated in the switching dynamics analysis (Fig. 4a). In its deglycosylated form (‘+PNGase’), hCGβ exhibits a slowed upward motion compared with glycosylated hCGβ (‘–PNGase’ and ‘untreated’, see below). Although the protein incurs a loss in molecular weight during deglycosylation, the switching measurement reveals an increase in hydrodynamic friction. This indicates that the deglycosylated protein adopts a less compact folding state, which has also been observed in studies on the impact of glycosylation on peptide conformation^33^. (Note that two negative controls were investigated to ensure that the harsh chemical treatment during the deglycosylation process did not harm hCGβ: ‘–PNGase’ samples underwent the same treatment like ‘+PNGase’ samples but were not exposed to PNGase. No significant differences were observed between ‘–PNGase’ and ‘untreated’ samples.)

To analyse the effect of protein phosphorylation, His_6_-tagged ERK2 in its non-phosphorylated and its phosphorylated state (P) was bound to the same layer of NTA_3_-modified DNA in subsequent measurements. In between measurements, the layer was regenerated by NaOH-induced dehybridization and rehybridization with fresh NTA_3_-modified single-stranded DNA. The phosphorylation state of ERK2 is clearly evident in the markedly different downward motions (Fig. 4c). Analyses of the downward DR values of six different electrodes confirm that ERK2-P can be unambiguously discriminated from ERK2 (Fig. 4d). The faster attraction of the DNA–ERK2–P complex most likely results from a combination of conformational changes (compaction) and an increase in negative charge due to the two additional phosphate groups.

## Discussion

Measuring the switching dynamics of electrically actuated molecules on microelectrodes combines two powerful bioanalytical concepts. On one hand, as the probe molecules are tethered to a surface and report the binding of targets in real time, the scheme features the merits of conventional label-free biosensors: the analyte solution can conveniently be exchanged above the detection spot using microfluidic systems, while association and dissociation kinetics are followed simultaneously. On the other hand, the switching method draws valuable information about size and shape from the analysis of molecular dynamics. In this respect, it bears similarities to DLS, by which diffusion coefficients are evaluated from Brownian motion, and electrophoresis, by which the drift velocity in electric fields is measured. Compared with light scattering, however, the switching method features a significantly better measurement accuracy when dealing with small- or medium-sized proteins (<50 kDa) and uses a fractional amount of sample (DLS: >10^−9^ mol; switching: <10^−12^ mol in the incubation solution, 10^−18^ mol bound per electrode).

The switching method is very effective for detecting slightest changes in tertiary protein structure and even post-translational modifications. Phosphorylation and glycosylation states could be identified, and ~40 kDa proteins differing only a few kDa in their nominal molecular weight could be discriminated according to their switching dynamics.

In combination with the Lollipop model, the switching dynamics measurement can be used to quantitatively determine protein diameters with an accuracy of 0.3 nm. This was successfully demonstrated for proteins with diameters ranging up to approximately one half of the used DNA lever length (*D*_H,prot_<½*L*_DNA_=8 nm for 48 bp DNA). The binding of larger proteins, such as antibodies (*D*_H_>11 nm) or antibody complexes, can be detected with even higher sensitivity, but, not unexpectedly, when the attached protein heads become too bulky steric interactions set in and the experimental curves deviate from the Lollipop calculations, which prevents the quantitation of absolute diameters. In future experiments, longer DNA levers will be used to size large proteins. For small analytes, the switching method reaches its limit if the bound target molecule does not significantly extend the DNA lever, that is, if the friction coefficients of the DNA–protein complex and the bare DNA lever do not differ substantially. Although this implies that the method is not ideally suited for the direct detection of small molecules in the sub-kDa molecular weight range, small proteins like ubiquitin (Ub; 8.5 kDa, *D*_H_≈2 nm) could be detected and sized successfully.

In spite of the fact that the switching is driven by electrical interactions between the charged DNA–protein complex and the biased surface, the influence of the protein charge was insignificant in the presented measurements. This counterintuitive finding is rationalized by the Lollipop model and also numerical simulations^34^: owing to counterion screening effects, the contribution of the protein charge to the total electric interaction strength rapidly diminishes within the scale of the Debye length (<2 nm) as the protein moves away from the surface. Therefore, the protein charge does not strongly affect the switching curve during most of its time-course. Instead, the protein charge is better observable in steady-state experiments, where, integrated over a couple of seconds, the equilibrated orientation of the protein–DNA complex is measured as a function of applied voltage. Preliminary results suggest that slight changes in the average DNA orientation due to repulsive or attractive contributions of the protein charge can be detected with high precision. We envision further exploiting this scheme for determining protein pI values on-chip.

As for any surface biosensor, it is important to discriminate non-specific interactions of target proteins with the sensor surface from specific probe–target interactions. In the case of electrically switchable DNA surfaces non-specific interactions may involve proteins adsorbing to the surface between the DNA, or proteins directly sticking to the DNA levers. The adsorption of proteins between the DNA levers is expected to lead to crowding, which should make it impossible for the DNA levers to lie down on the surface when applying attractive potentials (higher fluorescence at positive bias). Direct adsorption of proteins onto the DNA levers is expected to slow their motion; this ‘false-positive’ signal can be identified by comparison with negative controls (DNA molecules without capture molecules). In the present study, indications for non-specific protein interactions were not observed.

Resolving the switching dynamics of proteins bound to electrically actuated DNA levers provides quantitative information about protein size and structure. In combination with the possibility to array thousands of microelectrodes on one chip, and the ability to measure binding kinetics in real-time, electro-switchable interfaces have great potential as a versatile measurement modality for the simultaneous detection and analysis of proteins on a chip. This will help in eliminating the need to run multiple interaction and protein characterization assays sequentially, and facilitate improved workflows in drug discovery and protein engineering.

## Methods

### Chip and DNA layer preparation

The biochip consists of a glass substrate (27 × 40 mm) with eight holes (1 mm diameter), which serve as in- and outlets for four flow channels. Au work electrodes (120 μm diameter) and Pt counter electrodes were arranged in four areas with six electrodes each and fabricated by standard optical lithography and metallization techniques. Before DNA immobilization, the surface was cleaned in freshly prepared Piranha solution (95% H_2_SO_4_:30%H_2_O_2_=2:1) for 15 min, followed by extensive rinsing with deionized water, 3 min sonication and drying with nitrogen.

Thiolated 48mer oligonucleotides were end-grafted to the gold electrodes via spotting with a picolitre dispensing system in immobilization buffer (10 mM Tris pH 7.4, 200 mM NaCl, 1 μM DNA). The sequence of the Cy3 labelled single-stranded DNA for immobilization was 5′ HS-(CH_2_)_6_-TAGTCGTAAGCTGATATGGCTGATTAGTCGGAAGCATCGAACGCTGAT-Cy3 3′ (Metabion, Germany). After 10-min incubation, the chip was assembled by using double adhesive film with die-cut flow channels as an intermediate layer and a cover slide as a top layer. The flow channels were 60 μm high and 1 mm wide, and covered one of the four electrode areas each. The DNA-modified Au electrodes were passivated and unspecifically bound DNA was removed by coadsorbing mercaptohexanol (1 mM in ‘T’-buffer: 10 mM Tris pH 7.4, 50 mM NaCl) for 30 min (ref. 35). We note that the properties of the passivation layer with respect to long-term stability and protein repellence can be improved by employing longer alkane chains^36^, ternary^37^, or, for example, molecules with PEGylated head groups. After installation in the setup, the DNA layers were hybridized with complementary DNA and the density was adjusted using a previously reported electrical desorption procedure^38^. Briefly, the relative switching amplitude ΔF/F (ΔF is the observable fluorescence modulation amplitude when applying repulsive or attractive potentials, and F is fluorescence at repulsive potential) is used as a parameter that indicates the free mobility of DNA strands on the surface. Because of steric interactions, densely packed DNA levers cannot lie down on the surface, that is, cannot be switched (ΔF/F≈0%), while ultra-low-density layers feature ΔF/F values close to 100%. By applying negative voltage pulses (for example, −0.8 V versus Pt) for a couple of seconds, DNA was electrically desorbed from the surface until ΔF/F became maximal, indicating free DNA movement in a very dilute layer (density estimation <10^10^ molecules per cm^2^).

When required, double-stranded DNA layers were denatured by flowing NaOH solution (pH 12–13) over the electrodes for 10 s. The layers could then be regenerated by hybridization with fresh cDNA (50 nM in T-buffer, Supplementary Fig. S7). A flow rate of 100 μl min^−1^ was typically used during sensing experiments, which was high enough to operate the sensor in the reaction-limited kinetics regime. An effect of the applied hydrodynamic flow on the DNA switching behaviour was not observed.

### Modification of DNA with capture molecules and proteins

NTA_3_-tagged 48mer DNA was synthesized from triaminated DNA (IDT, Leuven, Belgium) according to the protocol of Goodman *et al.*^39^; the detailed procedure is described elsewhere^24^. Before each Nickel loading step, other divalent ions were removed by flowing a 5-mM EDTA solution in T-buffer over the sample for 10 min. After reducing the EDTA concentration to 50 μM, NTA_3_-tagged cDNA was loaded with 500 μM NiCl_2_ in T-buffer for 10 min. Afterwards, excess Ni^2+^ ions were washed out by thoroughly rinsing the surface with buffer solution.

MTX-modified DNA conjugates were prepared by activating the carboxyl groups of MTX through the incubation of 15 mM MTX with 60 mM EDC and 30 mM NHS for 10 min. The activated MTX was then added in 400-fold molar excess to 5′-NH_2_-modified cDNA (Metabion, Germany) in pH 7.4 PBS. After overnight incubation, MTX DNA conjugates were analysed and purified by RP-HPLC (Hydrosphere C18, YMC, Germany), followed by centrifugation with Amicon centrifugation filter units (Merck Millipore, Germany).

Protein A (*S. aureus*, Sigma Aldrich, Germany) was covalently conjugated to cDNA via lysines according to the following protocol: 5′-Thiol modified cDNA (Metabion, Germany) was reduced with TCEP (Tris-(2-carboxyethyl)phosphine, 100 mM, from Roth) for 30 min in 50 mM sodium phosphate buffer (pH 7.2, 150 mM NaCl) and purified by RP-HPLC (Hydrosphere C18, YMC). cDNA was modified by 6-Maleimidohexanoic acid *N*-hydroxysuccinimide ester using the manufacturer’s protocol (Pierce, Germany). After purification of the modified DNA from excess linker via Amicon centrifugation filters (Merck Millipore), protein A was mixed with DNA in a ratio of 2:1. The protein–DNA conjugate was purified by anion exchange chromatography and the 1:1 coupling stoichiometry was confirmed by ultraviolet absorbance and SDS–PAGE.

Cytochrome *c* (*S. cerevisiae*, Sigma Aldrich) has a free cysteine at the carboxy terminus, which was used for maleimide conjugation. 5′-amino modified cDNA (Metabion) was modified by 6-Maleimidohexanoic acid *N*-hydroxysuccinimide ester using the manufacturer’s protocol (Pierce). After purification of the modified DNA from excess linker via Amicon centrifugation filter units (Merck Millipore), cytochrome *c* was mixed with DNA (both 11 μM). The protein–DNA conjugate was purified by anion exchange chromatography and the successful 1:1 conjugation was determined by ultraviolet absorbance and SDS–PAGE.

Covalent conjugates of human carbonic anhydrase 1 (CA) and cDNA were prepared using hydrazine-aldehyde chemistry. Before use, CA was dissolved in pH 7.4 PBS and filtered using 0.2 μm syringe filters (Merck Millipore) to a final concentration of 1 mg m^−l^. Succinimidyl-6-hydrazino-nicotin-amide (Solulink, USA) in *N,N*-dimethylformamide (DMF) was added to CA at a tenfold molar excess, resulting in approximately one hydrazide group per CA. Separately, succinimidyl 4-formylbenzoate (Solulink) in DMF was added at a 20-fold molar excess to 5′-amino modified cDNA (IBA, Germany) in pH 7.4 PBS, resulting in the complete reaction of all 5′-amino groups to aldehydes within 3 h at room temperature (RT). Excess succinimidyl-6-hydrazino-nicotin-amide and succinimidyl 4-formylbenzoate were removed and the sample buffer was exchanged to pH 6.0 PBS using protein desalting spin columns (Pierce) and Amicon centrifugation filters (Merck Millipore), respectively. A twofold excess of derivatized CA was then mixed with cDNA and allowed to react overnight at RT. The conjugation product was purified by native PAGE in TBE buffer. CA–DNA monoconjugates were extracted from the gel by electroelution using D-Tube Dialyzers (Merck Millipore). The successful synthesis of CA-DNA 1:1 conjugates was additionally verified by absorbance and SDS–PAGE.

For conjugation of hCGβ to cDNA, 24 μl of a 1 mM solution of 6-Maleimidohexanoic acid *N*-hydroxysuccinimide ester in DMF was mixed with 2 nmol of reduced 5′-Thiolated c-DNA and incubated at RT for 45 min, followed by a buffer exchange into buffer A (50 mM sodium phosphate pH 7.4, 150 mM NaCl). For DNA–protein conjugation this solution was added to hCGβ in buffer A yielding a molar protein to DNA ratio of 2:1 and the reaction mixture was incubated at RT for 2 h. hCGβ conjugates were purified using anion exchange chromatography (AKTA purifier and Mono Q 5/50 GL column; GE Healthcare). The purity of the conjugate was confirmed by SDS–PAGE under reducing conditions.

### Time-correlated single photon counting setup and DNA switching potentials

Fluorescent light from the Cy3-labelled DNA levers was excited by a green LED (517 nm bandpass filtered) that was coupled into a × 50 objective via a dichroic mirror. Emitted Cy3 fluorescence light (570 nm) was collected by the same objective, passed through the dichroic mirror and a long-pass filter, and detected with a photomultiplier (Hamamatsu). Alternating voltage square-wave signals from a frequency generator were used to actuate the DNA molecules and simultaneously trigger an event timer of a single photon counting unit (NanoHarp; PicoQuant, Germany) to record the photon arrival time with 32 ns resolution. A Peltier element integrated in the sample holder enabled temperature controlled experiments.

Before every TR fluorescence measurement, the static fluorescence response to an applied voltage ramp was recorded (Supplementary Fig. S8). The switching potentials for TR measurements were chosen to be close to the two ‘plateau regions’ of the fluorescence response (indicating completely lying and completely standing DNA). The voltage amplitude was between 0.6 V and 1 V, the DC offset (≈0.0 V) was adjusted (app. ±0.1 V) according to the ‘potential of conformation transition’, that is, the inflection point in the measured voltage response curve. The frequency of the applied square wave potential was 10 kHz, which ensured that the DNA had enough time to stand up and lie down completely before reversing the voltage.

### Dynamic response

The DR is calculated from the normalized fluorescence signal *F*_norm_ as


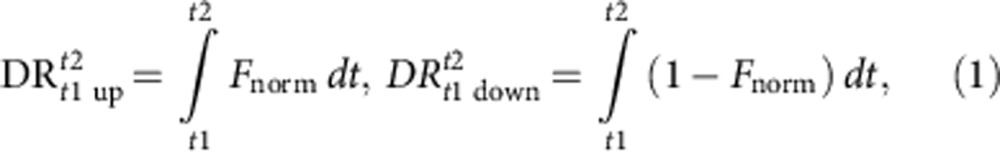


for the upward and the downward motions, respectively.

### Proteins for sizing measurements

Human DHFR was obtained from Sigma-Aldrich. His_6_-tagged proteins G and L were obtained from BioCat, Germany. His_6_-interferon-α and His_6_-Ub were provided by Qiagen, Germany. His_6_-tagged CaM and His_6_-tagged chloramphenicol acetyl transferase were obtained from Merck, Germany. All His_6_-tags were amino terminal. Antibiotin monoclonal mouse IgG was obtained from Sigma-Aldrich, antibiotin IgG fragment (Fab) from Rockland, USA.

### Determination of hydrodynamic diameters by DLS and XRD

Dynamic light scattering (DLS) experiments were conducted in 10 mM Tris pH 7.4 solution, using the Zetasizer Nano ZS instrument (Malvern Ltd, UK). Protein solutions (concentrations 10–100 μM) were filtered using Anotop syringe filters with a pore size of 20 nm. All measurements were conducted in a disposable capillary cell (DTS1061, Malvern Instruments, UK) that was carefully cleaned with isopropanol, filtered DI-water and dried under a stream of nitrogen. Before measurement, the temperature was equilibrated to 25 °C. Hydrodynamic protein diameters were obtained by fitting the autocorrelation function with a distribution of exponential decays.

Proteins with diameters below 5 nm could not be measured by DLS due to problems with low signal intensities and contaminants, agglomeration due to high concentration, or simply excessive sample consumption. Thus, effective hydrodynamic diameters were calculated from X-ray diffraction (XRD) structure data. Protein dimensions (x, y, z) were measured from pdb entries with Pymol (The PyMOL Molecular Graphics system, Version, 1.5.0.4 Schrödinger LLC; (CA: 3LXE, Ub: 1UBQ, INFα: 1ITF, DHFR: 2W3M, chloramphenicol acetyl transferase: 1PD5, ERK2: 4GT3). Effective hydrodynamic protein diameters were calculated according to Bloomfield^40^ assuming a prolate ellipsoid protein shape and using the geometrical mean of the two short axes as the short ellipsoid axis.

### Theoretical Lollipop model

Depending on the charging state Φ(*t*) of the microelectrode, the angle-dependent free energy of a rigid DNA lever with a (charged) protein attached to its distal end is given by


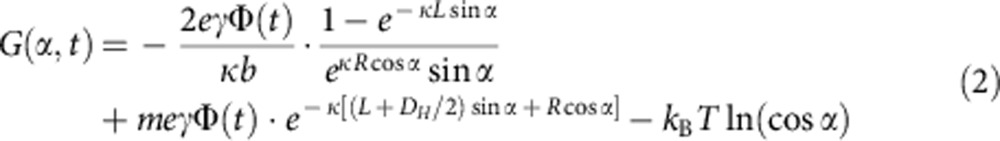


where *e* is the elementary charge, *γ* is an electrical screening parameter (0.016) that can be determined experimentally, *κ* is the inverse Debye length of the electrolyte solution, *b* is the base pair spacing (0.34 nm), *L* is the length of the DNA (16.32 nm), *R* is the hydrodynamic DNA radius (1.3 nm), *m* is the protein charge in multiples of the elementary charge, *k*_B_ is the Boltzmann constant, *T* is the temperature and Φ is the effective potential with respect to the surface’s potential-of-zero-charge. The time-variant electric potential Φ(*t*) of the microelectrodes is approximated by an exponential charging process


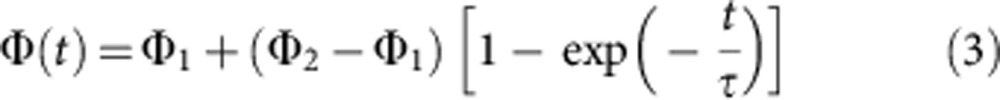


with *τ*=4*RC*/3 and *RC* being the measurable electrical charging time of the microelectrode. The factor 4/3 derives from an area weighing^41^. The non-equilibrium time evolution of the corresponding Boltzmann distribution is calculated numerically using a self-written Python code from the drift-diffusion Fokker–Planck equation:


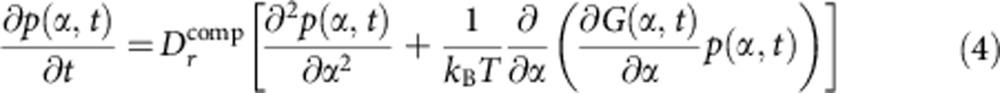


the probability distribution *p*(*α*,*t*) is converted to a fluorescence signal via


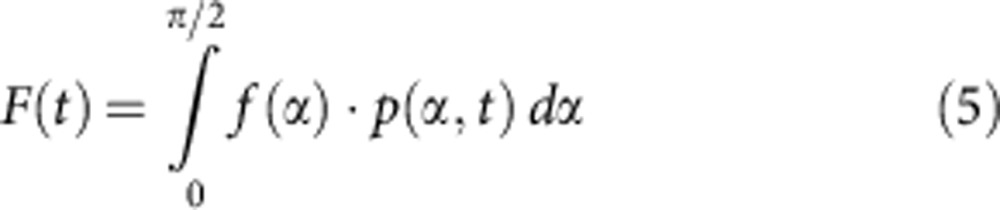


where *f*(*α*) is the known distance dependence of the fluorescence quantum yield^23^. The effective hydrodynamic protein diameter *D*_H_ is calculated by inverting the modified Einstein–Stokes equation^42^:


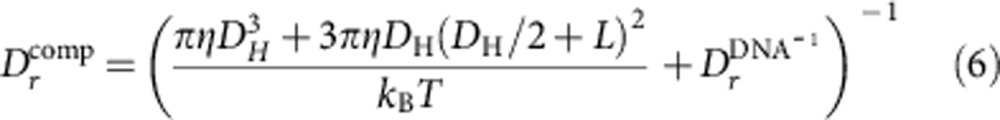


where *η* is the solution viscosity, and *D*_r_^comp^ and *D*_r_^DNA^ are the rotational diffusion coefficients of the DNA–protein complex and end-tethered 48 bp DNA (0.49 μs^−1^), respectively. *D*_r_^DNA^ was calculated with the formula of de la Torre and colleagues^43^.

### Unfolding of protein A

Urea and glycerol were purchased from Sigma. All solutions were prepared in 10 mM Tris pH 7.4 and 50 mM NaCl. Glycerol solutions of 3.5, 7, 10.5 and 14% (v/v) were used for the negative control.

### Post-translational modifications

For the phosphorylation of human extracellular signal-regulated kinase 2 (ERK2, Abcam, Cambridge, UK), 20 μg of His-tagged ERK2 were incubated *in vitro* with ERK kinase 1 (MEK1, Merck Millipore) at 30 °C for 30 min in phosphorylation buffer (0.7 μM MEK1, 9 mM Tris-HCl, 14 mM NaCl, 5 mM β-glycerophosphate, 1 mM EGTA, 0.2 mM sodium-ortho-vanadate, 0.4 mM dithiothreitol (DTT), 0.5 mM EDTA, 15 mM MgCl_2_, 0.1 mM ATP, pH 7.5). The successful phosphorylation was verified with phospho-ERK2-specific antibody (New England Biolabs, Germany) in a dot blot analysis (Supplementary Fig. S9).

The purified hCGβ–DNA conjugate was deglycosylated with PNGase F (NEB) according to the manufacturer’s protocol. For better deglycosylation efficiency the protein was denatured by incubation in glycoprotein denaturing buffer (6.2% DTT, 5% SDS; supplied by NEB) at 100 °C for 10 min. For deglycosylation the reaction mixture was supplemented with G7 reaction buffer (50 mM sodium phosphate, pH 7.5), 1% (v/v) NP-40 and 10% (v/v) PNGase F solution (all supplied by NEB) and incubated at 37 °C for 2 h. In order to eliminate the possibility that this procedure causes measurable changes to the protein structure that are not subject to deglycosylation one portion of the hCGβ-conjugate was treated in the described way but instead of PNGase F solution, water was added to the reaction mixture. To remove SDS and DTT a buffer exchange into buffer A was carried out. Successful deglycosylation was confirmed by SDS-PAGE under reducing conditions.

## Author contributions

P.H., W.K., J.K. and T.W. contributed equally to the manuscript. A.L, P.H., J.K., T.W., W.K., V.V. and M.M. conducted experiments. F.F., W.K. and P.H. developed the setup. R.S., T.W., V.V., A.L. and S.J. carried out chemical synthesis. M.S. and A.L. wrote the Python code. A.L. and U.R. wrote the paper. A.L., R.S. and U.R. devised research.

## Additional Information

**How to cite this article:** Langer, A. *et al.* Protein analysis by time-resolved measurements with an electro-switchable DNA chip. *Nat. Commun.* 4:2099 doi: 10.1038/ncomms3099 (2013).

## Supplementary Material

Supplementary Information Supplementary Figures S1-S9
