# Supplementary material for: Protein analysis by time-resolved measurements with an electro-switchable DNA chip
Source: Nat Commun. 2013 Jul 10;4:2099. doi: 10.1038/ncomms3099 (PMC3719012; doi:10.1038/ncomms3099)
Supplement: Supplementary Information — Supplementary Figures S1-S9 [file ncomms3099-s1.pdf]

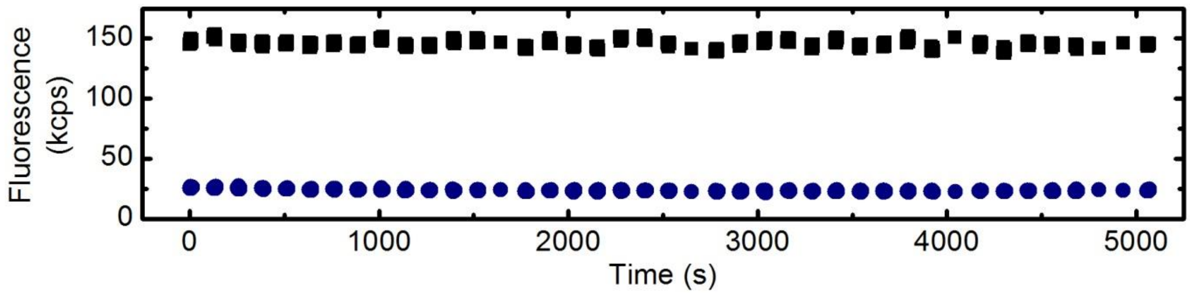

Supplementary Figure S1 | Long time switching. After switching of over 50 Mio cycles (app. 85 minutes), no loss of fluorescence is observable, indicating that neither DNA desorbed from the surface, nor Cy3-dyes were bleached. Black squares denote the fluorescence recorded at -0.2 V (standing DNA), blue circles are recorded at +0.5 V (lying DNA).

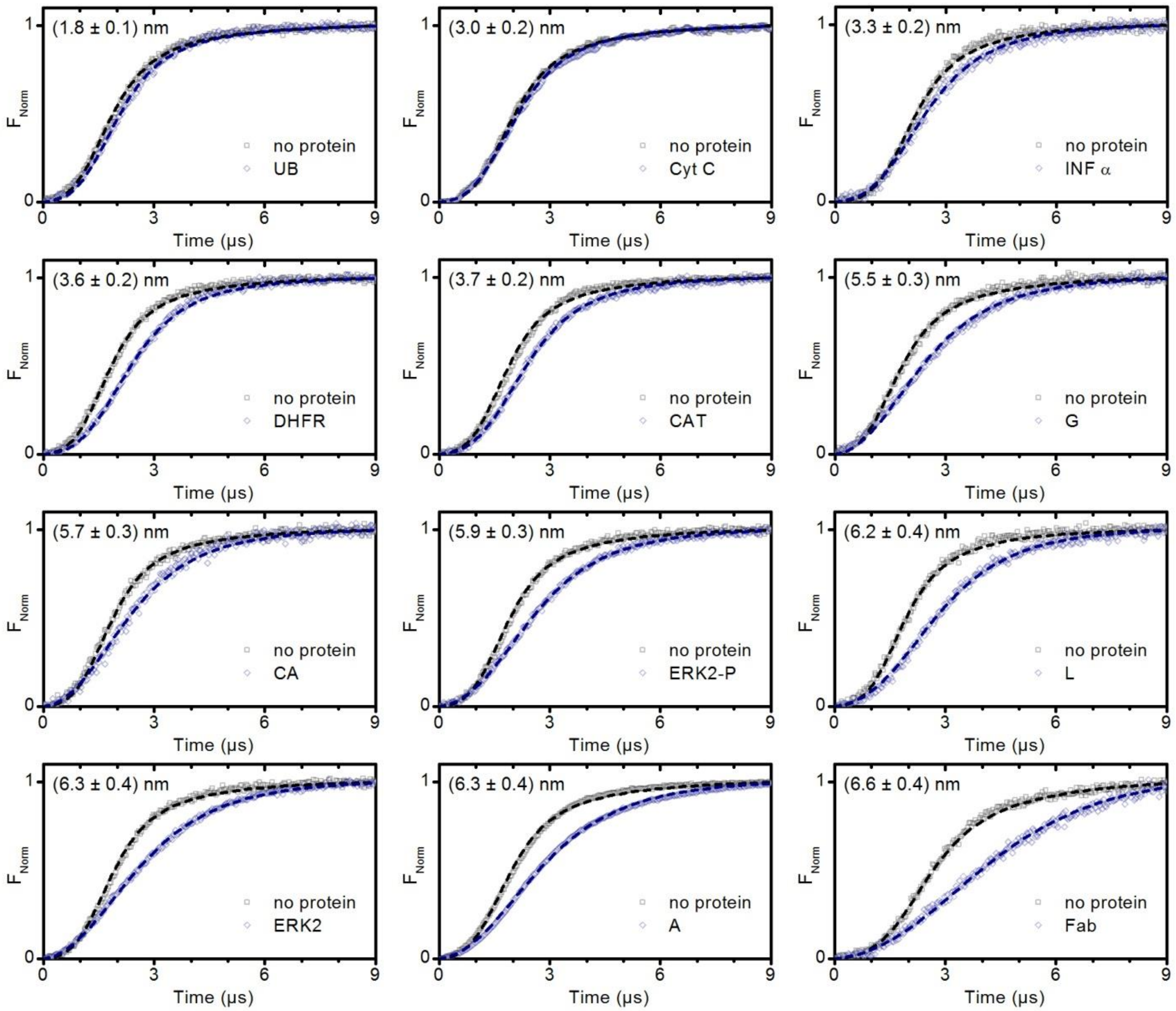

Supplementary Figure S2 | Upward switching fluorescence traces and Lollipop model fit curves. Numbers denote the effective hydrodynamic diameters obtained from the Lollipop model. Note that due to shifts in the potential-of-zero-charge before and after protein binding, as well as different choices of the switching potentials, smaller protein can show an apparent bigger shift than bigger proteins (i.e. UB and Cyt C). The theoretical model, however, takes the magnitude of the offset of the applied switching potentials into account and yields consistent size values.

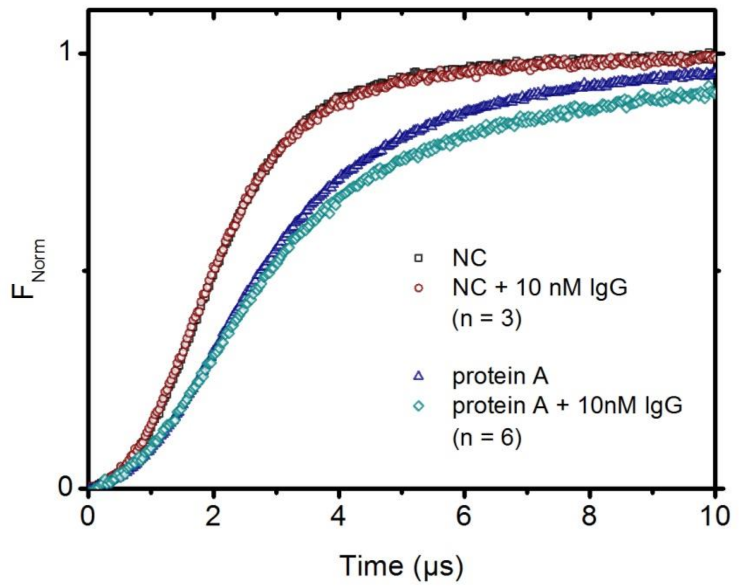

Supplementary Figure S3 | Binding of IgG to protein A modified DNA. Whereas the negative control (NC) shows no response to 10 nM IgG, the upward switching dynamics of a protein A modified DNA layer slow down due to specific binding of IgG to protein A.

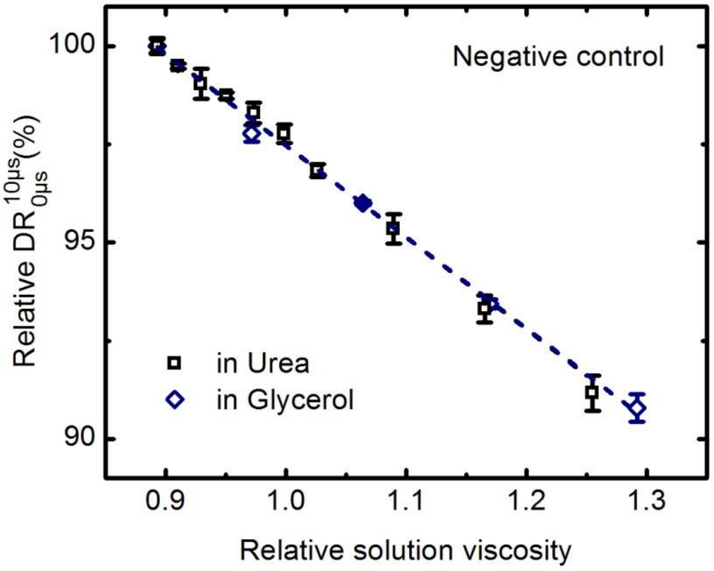

Supplementary Figure S4 | Unmodified DNA in urea and glycerin solutions. Due to a pure viscosity effect, unmodified DNA shows the same linear decrease of the Dynamic Response in urea as in glycerol.

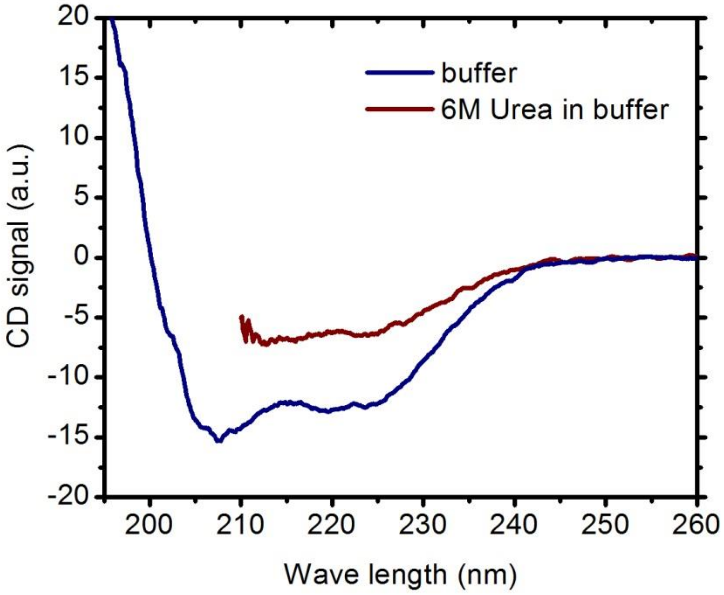

Supplementary Figure S5 | Circular Dichroism signal for native and denatured protein A. The biggest difference was found to be at 220nm. This wavelength was chosen for monitoring the conformational changes.

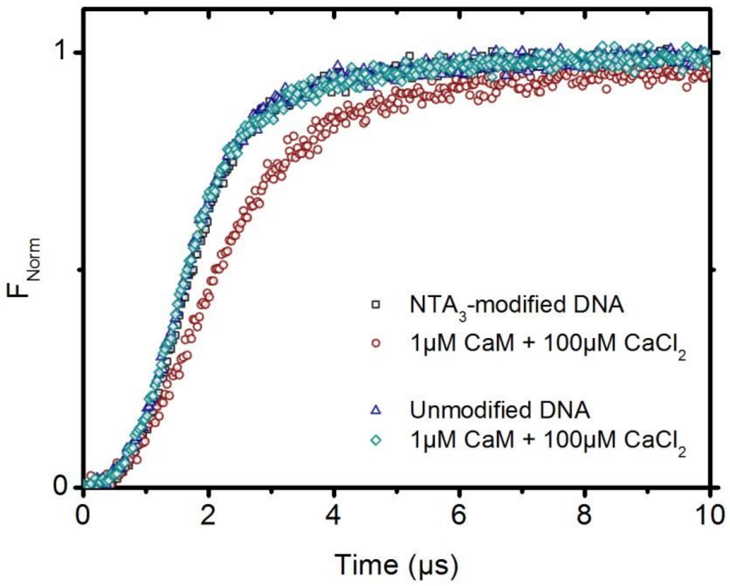

Supplementary Figure S6 | Binding of His$_6$-tagged Calmodulin (CaM) to NTA$_3$-modified and unmodified DNA. Upward switching dynamics for subsequent measurements on the same DNA layer: (1) NTA$_3$-modified DNA, (2) NTA$_3$-modified DNA in a solution of 1 µM CaM and 100 µM $CaCl_2$, (3) unmodified DNA, (4) unmodified DNA in a solution of 1 µM CaM and 100 µM $CaCl_2$.

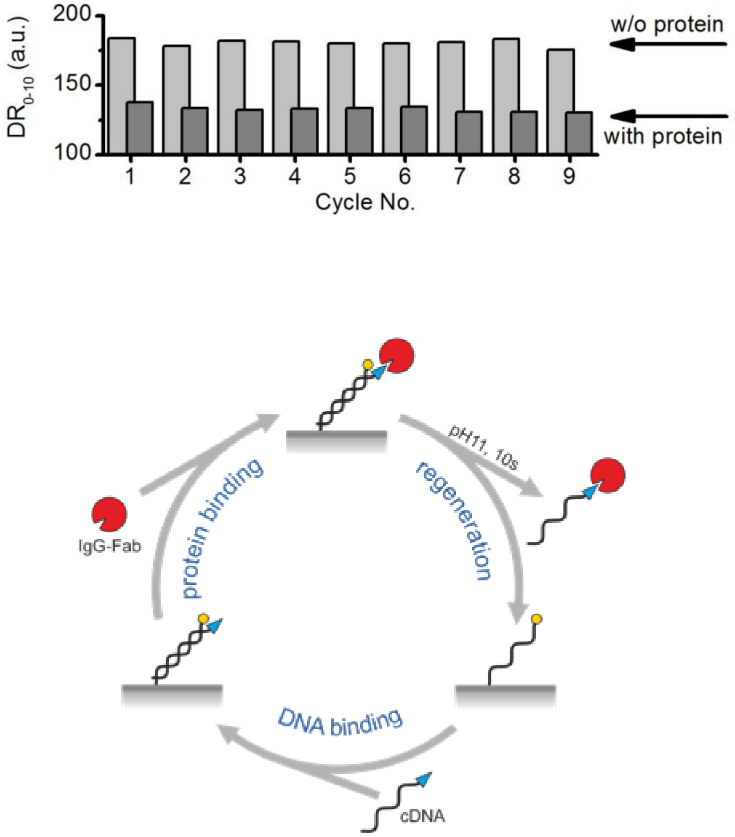

v

Supplementary Figure S7 | Layer regeneration. By NaOH induced dehybridization of the dsDNA, DNA layer can repeatedly be regenerated with fresh complementary, capture-molecule modified ssDNA. Analysis of the Dynamic Response shows that after each regeneration step, the same level of protein binding is obtained. The protein was IgG-Fab, the capture-molecule was biotin.

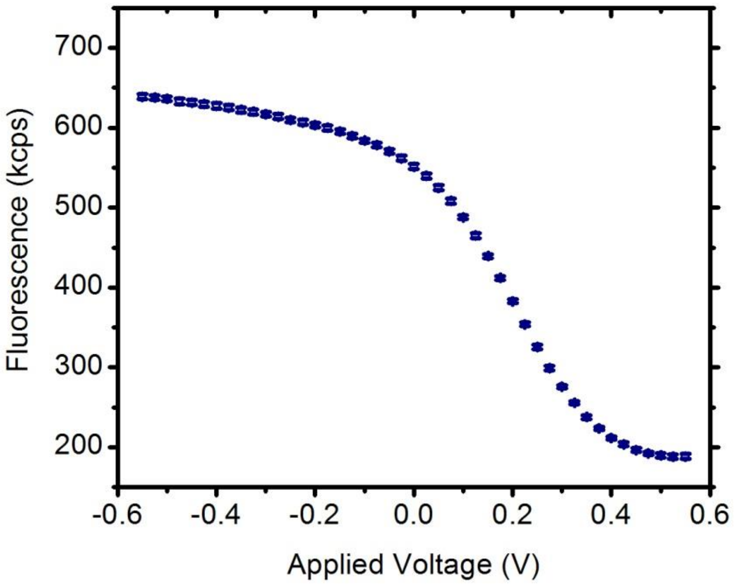

Supplementary Figure S8 | Static fluorescence response to an applied voltage. For high negative potentials, the fluorescence emittance is high: The DNA levers are repelled from the surface and the Cy3 fluorescence is less effectively quenched. For high positive potential, the fluorescence is low and saturates, indicating that the DNA levers are attracted to the surface and completely lie down. The Cy3 fluorescence is effectively quenched.

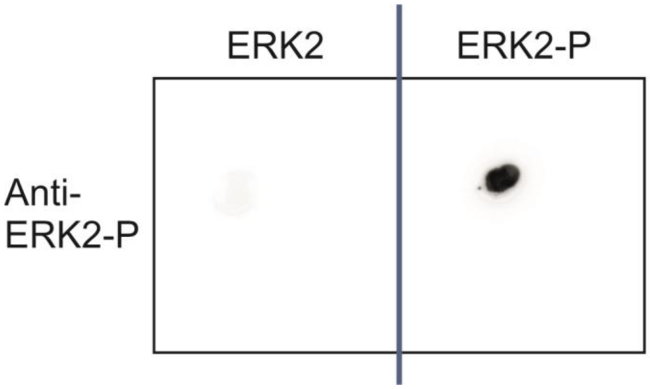

Supplementary Figure S9 | Phosphorylation of ERK2. A ERK2 Dot-Blot with a ERK2-P specific antibody shows the succesful phosphorylation of ERK2. 1 µg of ERK2 and 0.2 µg of phospho-ERK2 were spotted separately on a nitrocellulose membrane (Whatman, Hamburg, Germany) which had been activated by incubation in activation buffer (25 mM Tris pH 8.3, 20% methanol (v/v)) for 5 min. After 10 min incubation the membrane was blocked with 5% dried milk powder in PBST (140 mM NaCl, 2.7 mM KCl, 8 mM Na_2HPO_4, 0.05% Tween 20 (v/v)) over night at 4 °C. Subsequently, the nitrocellulose membrane was incubated for 2 h at room temperature with a phospho-ERK2 specific antibody (NEB, Germany; 1:2000 in blocking solution), followed by three washing steps at room temperature in PBST (10 min each). The secondary antibody (anti rabbit HRP-conjugate, NEB, Germany; 1:10000 in blocking solution) was incubated for 1 h at room temperature. Afterwards the membrane was washed as described before and specific protein binding was detected by enhanced chemiluminescence (Thermo Scientific, Rockford, USA).
